# The γH2AX DNA damage assay from a drop of blood

**DOI:** 10.1038/srep22682

**Published:** 2016-03-04

**Authors:** Daniel Heylmann, Bernd Kaina

**Affiliations:** 1 Institute of Toxicology, University Medical Center, Obere Zahlbacher Str. 67, D-55131 Mainz, Germany

## Abstract

DNA double-strand breaks (DSB) and blocked replication forks activate the DNA damage response (DDR), a signaling pathway marked by phosphorylation of histone 2AX (H2AX). The phosphorylated form, γH2AX, accumulates at the site of damage and can be detected as foci by immunocytochemistry. Therefore, γH2AX is a sensitive and robust biomarker of DNA damage, notably DSB. Cells from peripheral blood are often used for studies on genotoxic exposure of humans. They are limited, however, by the amount of blood required and the costly blood purification method. Here, we present a method that enables the detection of DNA damage by the analysis of γH2AX foci in a drop of blood. The blood drop method (BDM) is simple, fast, inexpensive and allows large series of blood sampling and storage over time. It can be combined with genotoxic treatment of cells in the collected blood sample for experimental purposes on DNA damage induction and repair. The BDM is suitable for rapid and large-scale screenings of genetic damage in human and animal populations.

Chemical mutagens, replication inhibitors, ultraviolet light, ionizing radiation and oncogenic transformation lead to replication stress and, directly or indirectly, to DNA double-strand breaks (DSB). These breaks represent the most severe form of DNA damage, since they result in chromosomal changes^1^ and cell death by apoptosis^2^. A complex signaling and DNA damage response network exists in order to detect DSB, maintaining genomic integrity and protecting against genotoxic effects^3^. Central players in this network are the PI3-like kinases ATM, ATR and DNA-PK which, upon activation, phosphorylate multiple substrates^4^. One of them is histone 2AX (H2AX), which becomes phosphorylated in the immediate vicinity of the break or at blocked replication forks^5^. Phosphorylation occurs at serine 139^6^, which is thought to alter the chromatin conformation and allows better access of repair enzymes to the damaged area^7^. Phosphorylated H2AX (designated as γH2AX) rapidly accumulates over megabase domains at the sites of DSB^8^ and, therefore, can be microscopically visualised as discrete nuclear foci. These foci can be detected by immunofluorescence using specific primary antibodies and secondary antibodies coupled with fluorescent dye.

There are other methods for quantifying DSB, e.g. the neutral comet assay^9^, pulse field gel electrophoresis^10^ and the TUNEL assay^11^. Although being constantly improved, these methods are quite laborious and insensitive for detection of low levels of DSB. Thus, the neutral comet assay delivers a significant signal at a dose >2 Gy (own unpublished data), i.e. above a damage level of about 80 DSB per cell^12^. In contrast, analysis of γH2AX foci allows DSB detection even in the mGy range, going down to a single DSB^13^. The number of intranuclear foci corresponds in a 1:1 ratio with the actual number of DSB^14^. Furthermore, co-localization of γH2AX foci with other repair proteins can be achieved, which enables mechanistic studies^15^. Thus, the high sensitivity and mechanistic understanding of γH2AX foci formation make them highly attractive as a biomarker for the presence of DNA damage.

Cells from peripheral blood are often used for biomarker screening, including chromosomal aberrations and other forms of DNA damage. Although peripheral lymphocytes are long-lived cells^16^, they undergo turnover and are constantly replenished from precursors in the bone marrow. Therefore, non-repaired damage in precursors and in lymphocytes can be considered as an outread for genotoxic exposures to a given individual. Usually, peripheral blood lymphocytes are purified by Ficoll gradient centrifugation^17^, for which a large quantity of blood (ml range) is required. Here, we report on a method that allows for γH2AX foci analysis from a drop of blood. Since blood collection and slide preparation is easy, Ficoll gradient centrifugation is not required and blood smears can be stored for several days at room temperature before further processing. The method paves the way for large-scale studies, screenings and routine investigations on damage to the human genome. We also show that the method can be extended to experimental settings and animal investigations.

## Results

**Figure 1:**
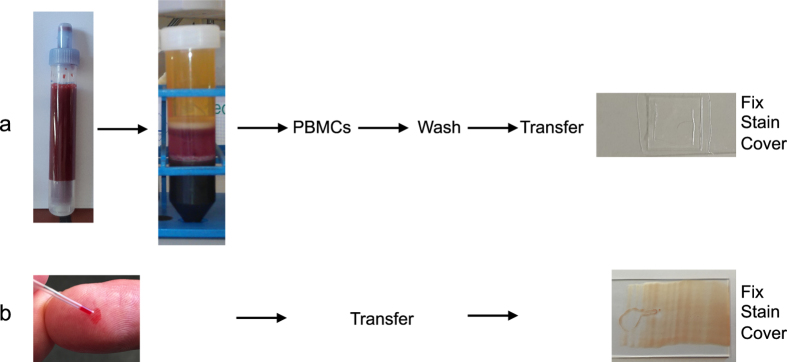
Blood for γH2AX staining. (**a**) PBMCs obtained by Ficoll gradient centrifugation. 5–10 ml peripheral blood were usually collected in heparinized tubes, which is layered onto a Ficoll gradient. Following centrifugation, the layer containing PBMCs was harvested, washed four times, concentrated by centrifugation and fixed onto glass slides. (**b**) A drop of blood is taken up in a heparinized glass micro-hematocrit capillary tube and spread onto one or several glass slides.

We compared γH2AX staining in peripheral blood mononuclear cells (PBMCs) obtained by Ficoll gradient centrifugation (Fig. 1a) and blood drop method (BDM). In the case of BDM, blood was obtained from the fingertip by means of a devise routinely used for diabetes testing and a disposable micro-hematocrit glass capillary tube (Fig. 1b). A drop of blood was sufficient for a smear on at least three (uncoated) glass slides. Alternatively, following punctuation, the drop of blood can also be taken up in a sterile micropipette and placed onto a glass slide. Using a second slide, the drop can be spread over the slide, covering an area of ~1 cm^2^. The blood is air dried at room temperature for ≥10 min before fixation in methanol/acetone or paraformaldehyde followed by immunostaining.

**Figure 2:**
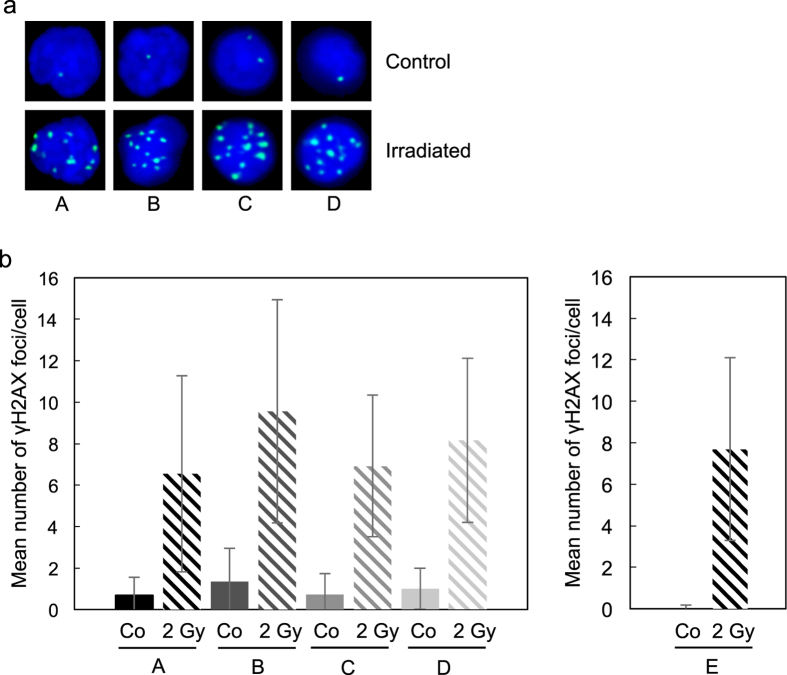
γH2AX staining and number of γH2AX foci in untreated (control) and irradiated blood cells. Irradiation occurred with 2 Gy followed by 60 min post-incubation at room temperature before immobilization of PBMCs on the slides. (**a**) Representative samples. (A) PBMC after Ficoll density centrifugation. (B) Whole buffy coat after erythrolysis. (C) Blood smear from buffy coat without Ficoll gradient purification and erythrolysis. (D) Lymphocytes from a drop of blood from fingertip prepared according to the BDM. (**b**) Number of γH2AX foci in control and irradiated cells. Following irradiation with 2 Gy, cells were post-incubated for 60 min, transferred to slides, fixed and stained. (A) PBMCs after Ficoll density centrifugation. (B) Whole buffy coat after erythrolysis. (C) Whole buffy coat without erythrolysis. (D,E) Drop of blood from fingertip. (A–D) counting occurred by means of the Metafer quantification system (automatic scoring using the imageJ software); (E) counting of γH2AX foci occurred visually using LSM. Error bars indicate the intraexperimental variation in the PBMC population.

We performed γH2AX staining using fresh drop blood slides and compared the result with PBMCs obtained in the conventional way from buffy coats. Buffy coats were subjected to Ficoll gradient centrifugation whereby PBMCs were separated from erythrocytes (Fig. 1a). Since erythrocytes are present in blood samples using BDM, we compared blood smears from buffy coats without and with erythrolysis by adding ammonium chloride. As shown in Fig. 2a (control), in non-irradiated samples lymphocytes were well recovered from the buffy coat without Ficoll gradient centrifugation and without erythrolysis. The background frequency of γH2AX foci/cell was nearly the same in Ficoll gradient purified lymphocytes in which erythrocytes were removed by centrifugation (Fig. 2a, panel A), without Ficoll gradient centrifugation, but with erythrolysis (panel B) and smear of buffy coat material without erythrolysis (panel C). The cells obtained by BDM (Fig. 2a, panel D) showed γH2AX staining similar to cells obtained from the buffy coat. The quantification is shown in Fig. 2b. This experiment revealed that lysis of erythrocytes is a non-essential step in PBMC preparation and γH2AX staining. The background γH2AX frequency of PBMCs on slides obtained by the conventional method and BDM was similar, i.e. ≤1 foci/cell.

Next, we wished to see whether BDM can be used to study the effect of radiation on peripheral blood. To this end, we irradiated PBMCs directly in the buffy coat or following purification by Ficoll gradient centrifugation and, thereafter, immobilized the cells on the slide. In parallel, we took up blood from a drop in a micro-hematocrit capillary tube and irradiated this sample *in situ*. One hour later, the cells were immobilised on a slide, air dried, fixed and stained for γH2AX. Representative examples are shown in Fig. 2a (irradiated) and the quantification is shown in Fig. 2b (columns lettered 2 Gy). The frequency of γH2AX foci was between 6 and 9 foci/cell and significantly enhanced above the non-irradiated control. The quantification was similar in BDM samples irrespective of whether quantification was performed by conventional light microscopy, using Metafer system and automatic scoring (as shown in Fig. 2b, panel D) or LSM (visually counted, shown in Fig. 2b, panel E). The data shows that irradiation of whole blood in a micro-hematocrit capillary tube *in situ* activates the DNA damage response (DDR), giving rise to γH2AX foci.

**Figure 3:**
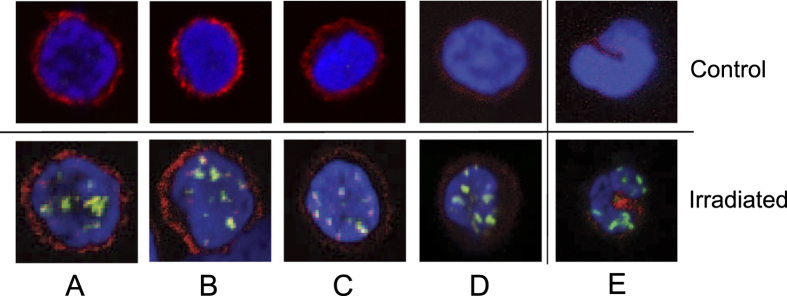
γH2AX in non-irradiated (control) and 2 Gy irradiated cells that were co-stained with CD3 for labeling T cells (**A–D**) and CD14 for labeling monocytes (**E)**. (**A**) PBMCs after Ficoll density centrifugation. (**B**) Whole blood from buffy coat with erythrolysis. (**C**) Whole blood from buffy coat without erythrolysis. (**D**) Drop of blood from fingertip. (**E**) Monocytes from drop of blood.

The blood cell subpopulations show a remarkable variability in their response to ionizing radiation^18^ and chemical genotoxins^19^, which explains the strong variability in the γH2AX foci number per cell observed in the whole blood samples (Fig. 2b). Therefore, in future applications, it will be important to determine γH2AX in specific blood cell sub-populations in order to assess their individual sensitivity and genotoxic response. Consequently, we set out to assess whether DNA damage determined by γH2AX can be measured in specific blood cell subpopulations using BDM, without FACS or other purification steps. As shown in Fig. 3, specific subpopulations of blood cells obtained by Ficoll gradient centrifugation or by BDM can be identified by immunohistochemistry, including CD3+ T cells (panels A to D; control and irradiated PBMCs, purified in the same way as in Fig. 2) and CD14+ monocytes (panel E). The staining quality of the surface marker was similar in Ficoll gradient purified samples and specimens obtained by BDM. This shows that it is possible to determine the basal and radiation induced γH2AX level in specific blood cell subpopulations using BDM, without lymphocyte purification and cell separation.

**Figure 4:**
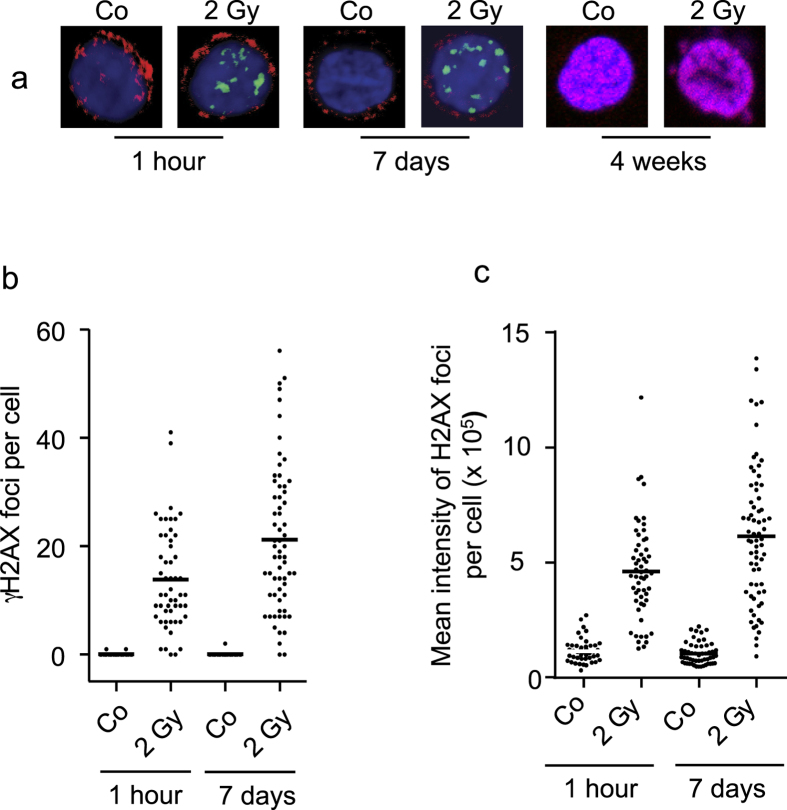
DNA damage in PBMCs obtained by BDM and irradiated in the micro-hematocrit tube. (**a**) Representative examples (Z-stacks) of CD3+ T cells of non-irradiated (Co) and irradiated (2 Gy) cells. Blood smears were prepared 60 min after irradiation on slides and air-dried. Staining for CD3 (red surface marker) and γH2AX (green) occurred immediately thereafter (1 hour samples) or 7 days or 4 weeks later with slides stored at room temperature in the dark. (**b**) Quantification of γH2AX foci in specimens stained 1 h or 7 days after immobilization on slides. γH2AX foci were visually counted. (**c**) γH2AX foci intensity was measured by integrated signal quantification. Imaging was performed by LSM.

How long can the slides be stored until they are subjected to immunostaining? We stained for γH2AX either 1 h or 7 days after slide preparation, using slides stored at 5 °C or room temperature after performing the blood smear. The γH2AX staining intensity in control and irradiated samples were rather similar comparing 1 h and 7 d slides (Fig. 4a for representative stainings; Fig. 4b for quantification of γH2AX foci/cell and Fig. 4c for intensity of γH2AX staining/cell). The stainings obtained with blood smears stored at 5 °C or room temperature (in the dark) were similar. However, after 4 weeks of storage at room temperature γH2AX staining was no longer possible and also CD3 immunostaining failed completely (Fig. 4a for representative staining). Importantly, air dried slides should be stored without fixation since fixation by methanol/acetone followed by paraformaldehyde worsened the staining intensity in 7 day stored slides. The same is true for CD3 staining and for γH2AX in irradiated samples. The staining quality was optimal if the slides with air-dried cells obtained by BDM were stored (up to 7 d) without any fixation. In conclusion, the method allows for large-scale studies with slide-immobilized cells from a drop of blood, upon storage for at least one week at room temperature.

**Figure 5:**
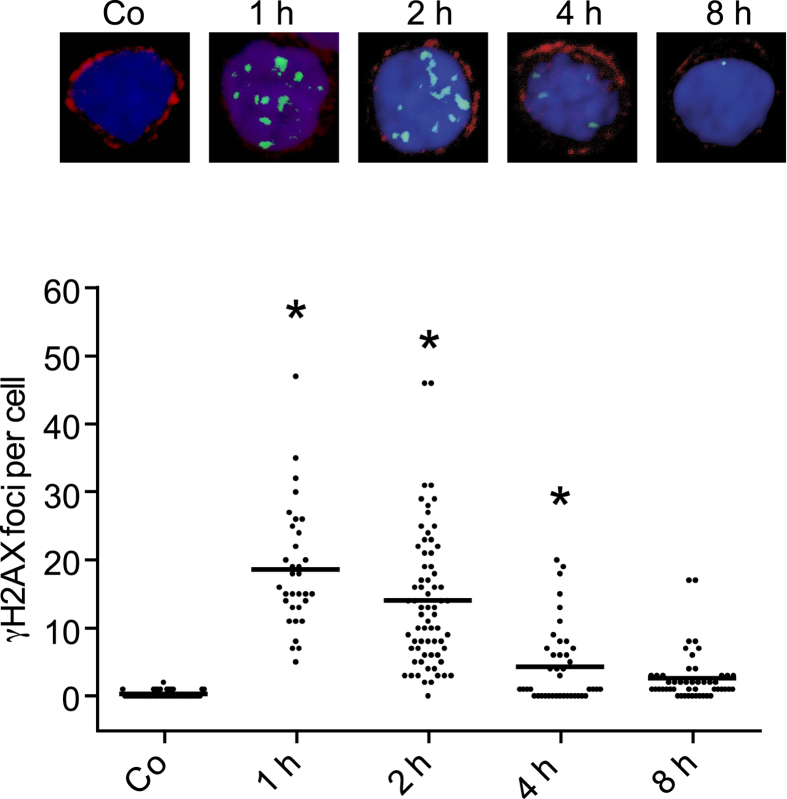
Human blood from the fingertip was collected in sodium-heparinized micro-hematocrit capillary tubes and irradiated in the tubes with 2 Gy. The tubes were incubated in sealed 15 ml Greiner tubes at 37 °C for the indicated time periods. Thereafter, blood smears were performed and stained for the CD3 marker and γH2AX. Imaging was performed using LSM (Z-stacks). The nucleus is stained blue, CD3 T cell receptor red and γH2AX green. The spot distribution shows the large variability of γH2AX in T cells. Asterisk indicates significant difference (p <0.05) to the non-irradiated control (Co).

Is BDM applicable for measuring DNA repair kinetics *in situ*? In order to answer this question, we used fresh blood from a healthy donor collected by BDM in micro-hematocrit capillary tubes and irradiated the tubes with 2 Gy. Several hours later, blood smears were performed, air dried and stained for the T cell marker CD3 and at the same time for γH2AX. As shown in Fig. 5, the yield of γH2AX foci in the T cell population was high 1 h after irradiation and dropped to nearly control level 8 h after irradiation. The half life of γH2AX was about 3 h (Supplement Fig. S1), which resembles the repair kinetics of DSBs in PBMCs reported previously^20^. Thus, the blood drop method allows for performing repair experiments, using small amounts of fresh blood samples obtained from donors without blood cell separation.

**Figure 6:**
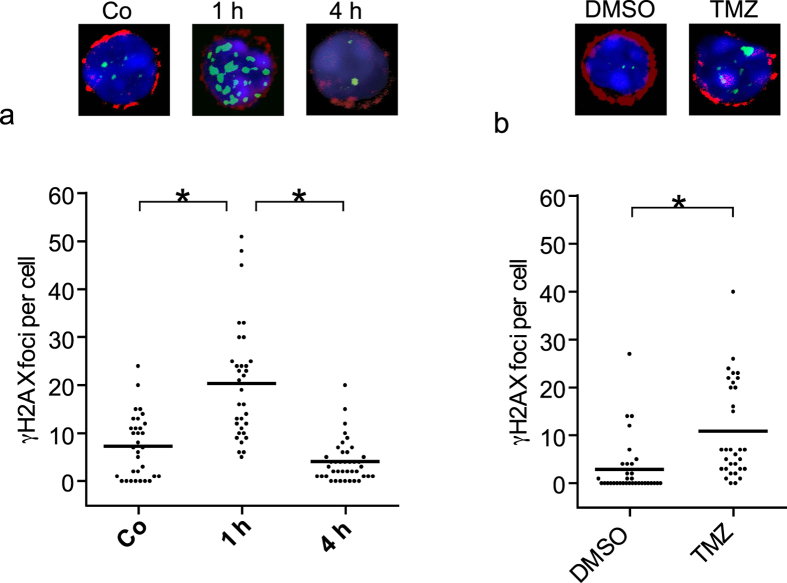
DNA damage in mouse T cells. (**a**) A drop of blood from mouse tail was collected in sodium-heparinized micro-hematocrit capillary tubes and irradiated *ex vivo* with 2 Gy. The tubes were incubated at 37 °C and blood smears were performed 1 and 4 h later. After air drying, they were stained for the CD3 T cell receptor and γH2AX. Imaging was performed using LSM. Representative pictures are shown (Z-stacks). The nucleus is stained blue; the CD3 T cell receptor red and γH2AX green. Asterisk indicates significant difference to the control and 4 h measure points (p<0.05). (**b**) C57 BL/6 mice were treated i.p. with 200 mg/kg TMZ or DMSO (solvent control) and blood was collected 16 h later for blood drop method. There is a significant increase of DNA damage (p < 0.05) in CD3+ T cells if mice were treated with temozolomide compared to the DMSO control. In the pictures on the top, the nucleus is stained blue, CD3 T cell receptor red and γH2AX green.

Next, we set out to determine whether the method is applicable for studies on animals, for which only small amounts of blood are available in experimental settings. In Fig. 6, we stained blood smears obtained from a drop of blood from tail veins of mice. The blood was non-irradiated (control) and irradiated *ex vivo* in micro-hematocrit capillary tubes immediately after harvest. After 1 h and 4 h incubation of the tubes at 37 °C, slides were prepared and stained for CD3 and γH2AX. It is shown that already 4 h after irradiation, the foci level is similar to the control, indicating ongoing DSB repair in mouse T cells (Fig. 6a). The method can also be applied for detecting DNA damage following treatment of mice with chemical genotoxicants, an example is shown in Fig. 6b. Here, we treated mice with the alkylating anticancer drug temozolomide, harvested blood in a micro-hematocrit capillary tube and immobilized the cells by a smear onto a slide. Sixteen hours after temozolomide injection, a significant amount of γH2AX foci was detected in mouse T lymphocytes, demonstrating the reliability of the method.

**Figure 7:**
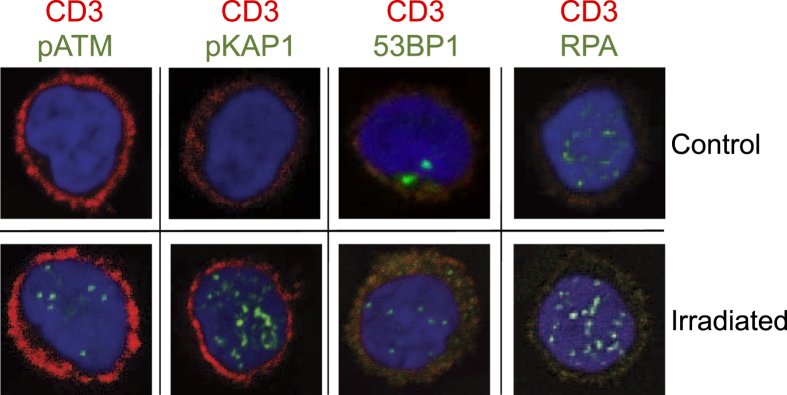
Staining of human T cells collected by BDM with pATM, pKAP1, 53BP1 and RPA/pRPA (green foci). All cells were co-stained with the CD3 surface marker (red staining).

To determine whether BDM allows for staining of other DDR marker than γH2AX, we stained slides with pATM, pKAP1, 53BP1 and RPA (using an antibody detecting phosphorylated and unphosphorylated RPA), all of them were reported to be indicators of DNA damage^3^. As shown in Fig. 7, air dried slides obtained by BDM and irradiated *ex vivo* in micro-hematocrit tubes showed staining with these markers and also co-staining with CD3 was feasible. We should note that for RPA a significant amount of foci was found in control cells, which were, however, less intense than in the irradiated samples. We should also note that following co-staining for RPA the CD3 staining intensity was reduced, possibly due to unspecific binding of the RPA antibody to CD3 and RPA epitopes on immobilized T cells.

## Discussion

γH2AX is a sensitive and robust biomarker for exposures to radiation and chemical genotoxicants^21^ and an indicator for DSB and/or blocked replication forks resulting from non-repaired bulky lesions or disturbed DNA metabolism^22^. Cells in peripheral blood do not proliferate, unless they were activated during the immune response. Therefore, in non-replicating PBMCs, the presence of γH2AX foci very likely represents non-repaired DSB that occurred in PBMC precursor cells or in PBMCs directly during their life. Radiation-induced DSB are repaired in PBMCs, but a residual level of non-repaired DSB appears to persist until the DSB bearing lymphocytes are eliminated by cell death^23^. Therefore, γH2AX foci in PBMCs are a valuable biomarker for endogenous and exogenous genotoxic insults.

DNA damage in PBMCs has been determined using various methods, including chromosomal aberrations, micronuclei and the comet assay, which require freshly prepared and purified PBMCs and, for chromosomal aberrations, *ex vivo* lymphocyte cultivation. Immunocytochemistry with PBMCs is also generally performed with fresh blood that is subjected to Ficoll gradient purification. This involves laborious and time-consuming working steps, which hamper large-scale population studies. The method reported here overcomes these limitations. Using BDM, only a small amount of blood is needed, which can directly be transferred to a microscope slide. The slides with the air-dried blood can be stored for at least a week at room temperature before they are fixed and stained. Staining with γH2AX can be combined with CD3 staining, which allows for γH2AX screening specifically in T cells. It can also be combined with other markers such as CD14 (for monocytes), enabling damage detection in different blood cell subpopulations. The method is also applicable for stainings with other DDR markers that become phosphorylated in response to DNA damage, such as pATM^4^, pKAP1^24^, 53BP1^25^ and pRPA^26^. With these DDR players, co-staining with CD3 was achieved on slides prepared by means of BDM.

The method can also be applied for experimental purposes. Thus, blood taken up in a micro-hematocrit capillary tube can be irradiated and stored for several hours at 37 °C until fixation on slides. The γH2AX time course demonstrates that repair occurred with a similar kinetics as reported for lymphocytes irradiated *ex vivo*^20^. The method was also shown to be applicable for experiments with mice when only small amounts of blood are available. There is no reason that speaks against a general application for animal monitoring even under field conditions. The method is robust. It does not need a clean bench and an incubator, nor excessive laboratory equipment for lymphocyte purification and slide preparation. It is, therefore, a method that paves the way for large-scale studies on human (and animal) populations exposed to risk factors such as environmental pollution, ultraviolet and ionizing radiation (emitted on the ground and following high altitude travelling, space mission and therapeutic applications), dietary factors (including food carcinogens), lifestyle drugs and endogenous (inflammatory and emotional) stress. It may be applied for radiation-biodosimetry and assessment of the late effects of anticancer drugs. Moreover, it allows for an individualized screening for the presence of DNA damage in individuals who are at risk or just want to know their DNA damage status.

## Methods

### Isolation of human lymphocytes from buffy coat

PBMCs were isolated from human peripheral blood (leucocyte rich plasma - buffy coat) from healthy volunteers using the Ficoll-Paque density centrifugation. 35 ml of buffy coat were carefully layered on 15 ml Ficoll-Paque (Histopaque-1077, Sigma Aldrich, St. Louis, USA) in a 50 ml Greiner tube and centrifuged at 2,500 rpm for 40 min at 20 °C without break. Three layers can be separated, a plasma layer, the interphase containing the PBMCs (lymphocytes, monocytes) and a small layer of Ficoll-Paque. Erythrocytes and granulocytes are concentrated at the bottom. The interphase was transferred to a new 50 ml Greiner tube. The tube was filled completely with buffer (2 mM EDTA in PBS with 0.5% bovine serum albumine), mixed and centrifuged at 1,500 rpm for 10 min at 20 °C. Following this washing step, the supernatant was removed, the cell pellet resuspended in 50 ml buffer and centrifuged again at 1,200 rpm for 10 min at 20 °C. After removing the supernatant, the last steps were repeated twice at 900 rpm for 10 min to remove residual platelets. Finally, 5×10^5^ cells per 1 ml X-VIVO 15 (BioWhittaker, LONZA, Basel, Switzerland) medium were cultured in 15 ml Greiner tubes, irradiated with 2 Gy (γ-rays) and incubated at 37 °C for up to 60 min.

### Lysis of erythrocytes in buffy coats

5 ml of ammonium chloride lysis buffer (0.155 mol/l NH_4_Cl 0.01 mol/l KHCO_3_, 0.1 mol/l EDTA, in distilled H_2_O, pH 7.3) were mixed with ~100 μl buffy coat in a 15 ml Greiner tube. The cells were incubated for 20 s at room temperature to lyse the erythrocytes. Then, the tube was filled with buffer (0.5% albumin fraction V (BSA), 2 mM EDTA in PBS), carefully mixed and centrifuged at 1,200 rpm for 10 min. The supernatant was completely removed and the washing step was repeated. The cell pellet was re-suspended in 1 ml X-VIVO 15. Following irradiation with 2 Gy, the cell suspension was incubated at 37 °C for up to 60 min.

### Preparation of blood smear from buffy coat

A drop of irradiated or non-irradiated sample of buffy coat incubated at 37 °C for up to 60 min was placed on one end of a microscope slide. Another microscope slide was used to disperse the sample over the whole slide length. The slides were left on disposable staining trays for air drying for 10 min in the dark before fixation.

### Preparation of blood smear from the fingertip (BDM)

For receiving a drop of blood from the fingertip, a lancing aid sold commercially for diabetes testing was used. Donor of blood for all blood drop experiments was one of the authors (BK). The blood was taken up in a sodium-heparinized micro-hematocrit glass capillary tube (Marienfeld superior, Lauda Königshofen, Germany) (Fig. 1b) and immediately thereafter brought onto the slides. Alternatively, the drop can also be taken up in a sterile tip with a pipette and transferred onto the slides. For irradiation of blood samples *in situ*, blood was taken up from a drop in micro-hematocrit glass capillary tubes and stored in a 15 ml Greiner tube in a horizontal position. One capillary tube was irradiated while the other was left unirradiated (control). Thereafter, the glass capillary tubes were stored in a horizontal position in the Greiner tube for up to 60 min at 37 °C in an incubator. The blood was released on microscope slides by tapping and dried onto the slides for at least 10 min.

### Fixation and immunostaining for Laser Scanning Microscopy (LSM)

The Ficoll purified samples were washed in PBS and centrifuged at 1,500 rpm for 5 min at 4 °C. The supernatant was removed and the cell pellet was taken up in 3 μl PBS. 1 up to 3 μl of this cell suspension was placed onto dry cover slips, which were placed in 6 well plates. The cells were air dried for 1–3 min and permeabilized with methanol:aceton (7:3) solution for 6 min at −20 °C. The solution was removed and the slides were washed 2 times with PBS in 6 well plates. The samples were incubated with 2.5% paraformaldehyde for fixation (10 min at room temperature). The paraformaldehyde was removed and the slides were washed 2 times with PBS. Each slide was covered with 10% normal goat serum (Invitrogen, Life Technologies, Carlsbad, USA) in PBS for 30 min. Then the slides were co-incubated for 1 h with antibody against phospho S139 Histone H2A.X and antibody against CD3, each of them dissolved 1:100 up to 1:500 in 1% BSA in PBS. After washing the slides 3 times with PBS for 5 min, they were incubated for 1 h with goat anti-rabbit Alexa Fluor 488 (Invitrogen, A11070) 1:300 (for detecting γH2AX) and goat anti-rat Cy3 (Jackson Immuno Research, 112 165 143, Baltimore Pike, USA) 1:500 (for detecting CD3) in PBS solution containing 1% BSA. The slides were washed again with PBS and stained for 15 min with ToPro3 (Invitrogen, Oregon, USA) 1:100 solved in PBS. The slides were mounted in VECTASHIELD medium (Burlingame, USA) on microscope slides and sealed with nail polish. In all immunostainings, similar results were obtained when the γH2AX antibody was diluted up to 1,000-fold. The same was observed for the CD3 antibody as well as for the secondary antibodies, which allowed dilution up to 1,000-fold without impairing staining quality (see Supplement Fig. S2). The following antibodies were used for human cells: phospho S139 Histone H2A.X monoclonal from rabbit (Abcam 81299, ChIP Grade, Cambridge, UK), CD3 from rat (AbDSerotec MCA 1477, Kidlington, UK), phospho ATM (phospho S1981) from rabbit (Abcam, EP1890Y, ab81292), phospho KAP-1 (phospho S824) from rabbit (Bethyl laboratories.inc, A300-767A-T), 53BP1 from mouse (Millipore, MAB3802) and RPA antibody from mouse (Millipore, NA19C). For the combination of 53BP1 or RPA together with CD3 we used as a secondary antibody goat anti-mouse Alexa Fluor 488 (Invitrogen, A11017).

### Fixation and immunostaining for Metafer Slide Scanning System

The cells were fixed with 4% paraformaldehyde for 10 min at room temperature. After washing the samples with PBS, they were incubated with ice-cold methanol for 10 min at −20 °C. The samples were washed again with PBS and blocked with 10% goat normal serum + 0.1% Triton X-100 in PBS for 30 min. The samples were incubated with the antibodies as described above except that the solution contained additionally 0.1% Triton X-100. Slides were mounted with VECTASHIELD medium with DAPI and sealed with nail polish.

### Imaging systems

The LSM 710 (Carl Zeiss, Oberkochen, Germany) operated with the ZEN software was used for Laser Scanning Microscopy. Images of γH2AX foci were taken, counted visually or by measuring the mean intensity of the γH2AX signal. The mean intensity of γH2AX foci was detected by manually labeling the nucleus using the ImageJ software. Different cross-sections of a cell (called Z-stacks) were scanned by the LSM system and assembled in a three-dimensional picture. The Metafer slide scanning was performed with the Axio Imager M1 (Carl Zeiss) along with the Metafer4 software (MetaSystems, Altlussheim, Germany), which allows single-cell picture recordings. Automatic counting of γH2AX foci was performed by an appropriate batch-macro in ImageJ (Fiji). During this process, cells were screened automatically for γH2AX foci, which were listed in a separate window.

### Repair kinetics

Blood was collected in a sodium-heparinized micro-hematocrit capillary tube from a healthy donor (BK) as described above. The micro-hematocrit capillary tubes were placed in a 15 ml Greiner tube and non-irradiated or irradiated with 2 Gy using a GammaCell 2000 (Cs137). Thereafter, the sealed Greiner tubes were incubated at 37 °C for the indicated post-exposure times (up to 8 h) and slides were prepared as described and stained with CD3 and H2AX antibody.

### Mouse experiments

5 weeks old C57 BL/6 mice were treated *i.p*. with 200 mg/kg temozolomide (Schering-Plough, Whitehouse Station, NJ) dissolved in DMSO. Control mouse was treated with DMSO only. Blood was collected from tail/heart puncture and directly smeared on glass slides for fixation and staining. Blood from mouse collected in sodium-heparinized micro-hematocrit capillary tubes was irradiated *ex vivo* with 2 Gy γ-rays. Blood smears were performed and slides were simultaneously stained with CD3 antibody (AbDSerotec MCA 1477, Kidlington, UK, dissolved 1:100 in 1% BSA in PBS) and γH2AX antibody (Abcam 81299 ChIP Grade, Cambridge, UK, 1:100) at the times indicated. After being washed 3 times with PBS for 5 min, the slides were incubated for 1 h with goat anti-rabbit Alexa Fluor 488 (Invitrogen, A11070) 1:300 and goat anti-rat Cy3 (Jackson Immuno Research, 112 165 143, Baltimore Pike, USA) 1:500 in PBS containing 1% BSA.

### Statistics and ethic statement

Statistical one-way Anova analyses were run under Graphpad Prism. Samples were obtained with informed consent from all subjects. All experiments, including those with mice, were performed in accordance with guidelines and regulations of the Ethics Committee of Rhineland-Palatinate, and experimental protocols were approved by this institutional committee.

## Additional Information

**How to cite this article**: Heylmann, D. and Kaina, B. The γH2AX DNA damage assay from a drop of blood. *Sci. Rep.* **6**, 22682; doi: 10.1038/srep22682 (2016).

## Supplementary Material

Supplementary Information
