# Supplementary material for: The γH2AX DNA damage assay from a drop of blood
Source: Sci Rep. 2016 Mar 4;6:22682. doi: 10.1038/srep22682 (PMC4778029; doi:10.1038/srep22682)

**Supplement information**

**Manuscript title:**

The γH2AX DNA damage assay from a drop of blood

**Authors:**

Daniel Heylmann and Bernd Kaina


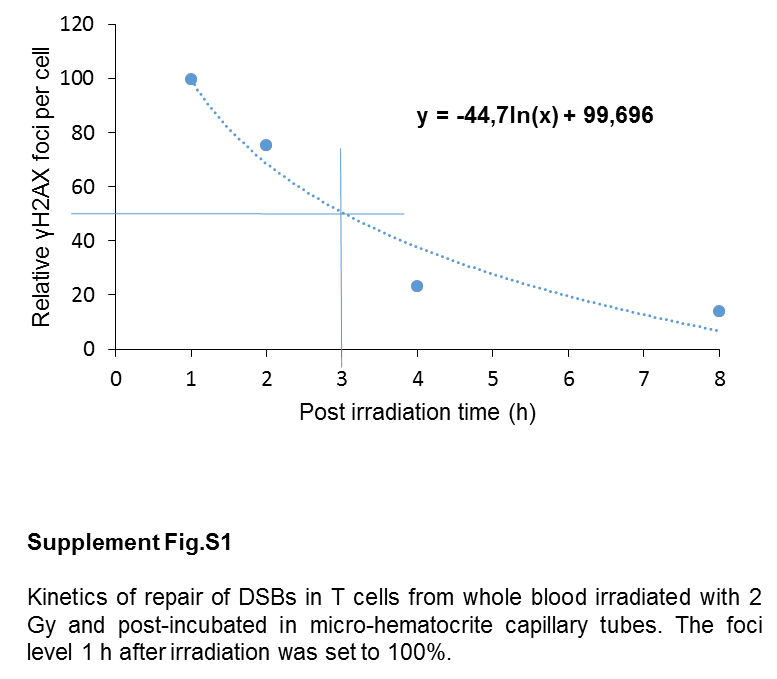


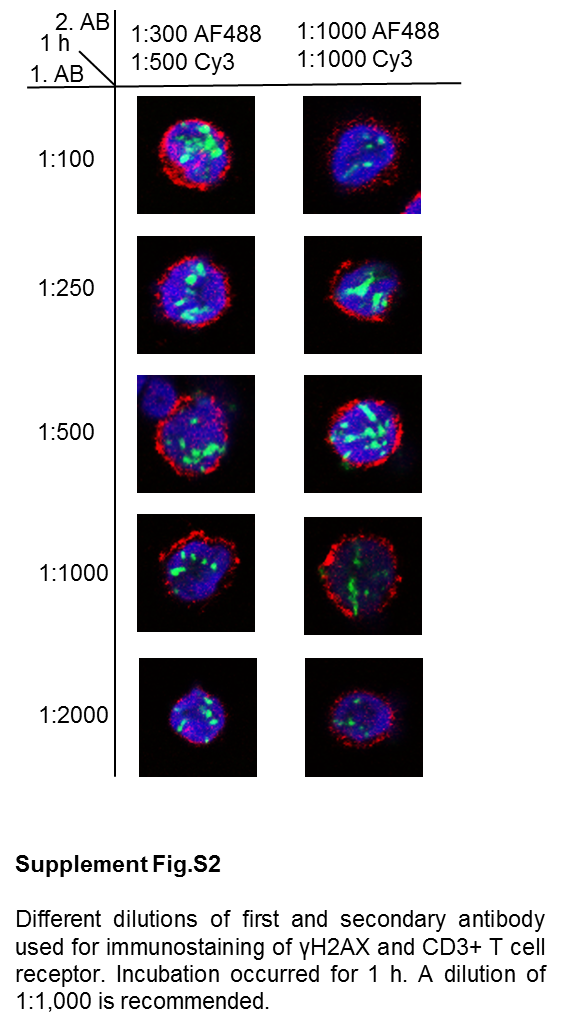

Supplement: Supplementary Information [file srep22682-s1.doc]
